# Where do the symptoms come from in depression? Topography and dynamics matter

**DOI:** 10.1093/braincomms/fcae067

**Published:** 2024-03-05

**Authors:** Yasir Çatal, Georg Northoff

**Affiliations:** The Royal’s Institute of Mental Health Research & University of Ottawa, Brain and Mind Research Institute, Centre for Neural Dynamics, Faculty of Medicine, University of Ottawa, Ottawa, Ontario K1Z 7K412, Canada; The Royal’s Institute of Mental Health Research & University of Ottawa, Brain and Mind Research Institute, Centre for Neural Dynamics, Faculty of Medicine, University of Ottawa, Ottawa, Ontario K1Z 7K412, Canada

## Abstract

This scientific commentary refers to ‘Brain dynamics predictive of response to psilocybin for treatment-resistant depression’, by Vohryzek *et al*. (https://doi.org/10.1093/braincomms/fcae049)

**This scientific commentary refers to ‘Brain dynamics predictive of response to psilocybin for treatment-resistant depression’, by Vohryzek *et al*. (https://doi.org/10.1093/braincomms/fcae049)**

##  

### From computational modelling to depressive symptoms

In their unique paper, Vohryzek *et al*.^1^ show that they can simulate the neural effects of psilocybin *in silico* neural models by being able to distinguish between responders and non-responders. For that, they use both global and local regional parameters in their whole brain model. The global parameters refer mainly to global connectivity strength as an index of global neural synchronization that, as their and other^2,3^ show, is abnormally high in especially default mode network (DMN) regions in acute major depressive disorder. While local measures concern the bifurcation index thus conforming to non-linear dynamics as well established in physics.

They point out a variety of regions including some lower order unimodal sensory regions and several higher order transmodal associative regions that distinguish responders and non-responders in both fMRI data and mathematical model. This is an amazing achievement as we all know that computational modelling of empirical data and especially those of fMRI is, to say the least, not an easy undertaking. The group around Deco and Kringelbach is indeed one of the world top groups if not the leading group in this emerging field of computational neuroscience and psychiatry. Even more important, as the authors themselves point out, this carries major clinical implications like the simulation of individual brain patterns prior to their possible therapy with psilocybin. For instance, based on their method, one may be able to determine, using simulation, whether a subject will respond neurally and subsequently also clinically to psilocybin or not. This could guide therapeutic application and thus provide therapeutic predictive markers that, currently, are lacking completely in clinical psychiatry.

This also raises a key question in the wake of their model. Where and how the depressive symptoms come from, we currently do not know. The authors themselves provide a hint. They show that their predictive regions map well with the raphe nucleus based subcortical-cortical projections. But why and how does that lead to the peculiar symptom constellations of depression where the often neglected somatic, arousal and psychomotor symptoms go along with major changes in mood, affect and cognition. We currently do not know. This is the moment where, taking one step further their global approach to the brain, one may want to look at subcortical-cortical topographic patterns.

### From subcortical-cortical topography and dynamics to the co-occurrence of symptoms

The cortical regions and networks are modulated by various subcortical nuclei and their biochemicals. These include the noradrenergic locus coeruleus (LC),^4^ the dopaminergic substantia nigra (DA-SN) and ventral tegmental area (DA-VTA), the acetylcholinergic nucleus basalis meynert (Ach-NM) and the serotoninergic raphe nucleus (SN-RN),^5-7^ respectively. Recent studies showed an opposite modulation of the sensorimotor network (SMN) and related movements by DA-SN and SN-RN. Increases in the resting-state functional connectivity (rsFC) from DA-SN to the SMN (through basal ganglia and thalamus) lead to increased activity in SMN resulting in increased movements.^5^ The opposite is the case in SN-RN whose increase in its rsFC to SMN (through modulation of the dopaminergic pathways) inhibits SMN activity leading to decreased movements.^5-7^ Hence, there is reciprocal modulation of cortical SMN and related psychomotor activity by the subcortical dopaminergic and serotoninergic nuclei and their pathways. This amounts to an intrinsic or systematic serotoninergic–dopaminergic subcortical-cortical SMN topographic relationship from which one can basically infer what happens when one or both of the biochemical neuromodulators, dopamine and serotonin, becomes deficient.

A series of resting-state fMRI subcortical-cortical functional connectivity (FC)^6-8^ extended these observations to bipolar disorder and schizophrenia. In manic bipolar disorder patients, DA-SN-based resting-state FC is reduced that leads to increased thalamus-SMN resting-state FC with psychomotor agitation (see also Martino *et al*.^7,8^ and Northoff *et al*.^9^) While in depressive bipolar disorder patients, reduction in DA-SN was accompanied by decreased SE-RN that together results in decreased thalamo-SMN rsFC with psychomotor retardation.^5,6^ We thus can see how subcortical DA-SN based on SE-RN imbalances of the intrinsic subcortical-cortical SMN topography leads to abnormal psychomotor activity, that is, increased or reduced.

Does this entail a cross-diagnostic dimensional symptom-based approach? Magioncalda *et al*.^6^ showed that all bipolar disorder and schizophrenia patients showing psychomotor agitation exhibited increased thalamus-SMN FC and reduction of RN-thalamus FC. While those subjects with psychomotor inhibition showed decreases in both thalamus-SMN FC and RN-thalamus FC. These and other studies^9,10^ supported a symptom-based dimensional cross-diagnostic subcortical-cortical correlate of psychomotor activity, e.g. agitation and inhibition.

How about depression in major depressive disorder? Some studies observed changes in SN-RN and DA-VTA in major depressive disorder. Anand *et al*.^11^ investigated medication-free young adult major depressive disorder subjects focusing on their subcortical-cortical fMRI resting-state FC. SE-RN FC to DMN regions in prefrontal and anterior cingulate cortex was decreased that, in amygdala and hippocampus, also correlated with major depressive disorder severity. In line with this, Wohlschläger *et al*.^12^ investigated the power spectral density of SE-RN and DA-VTA in unmedicated major depressive disorder. This study found a significant spectral slowing, e.g. shift of the power spectrum towards slower frequencies, with lower permutation entropy in DA-VTA and SE-RN in major depressive disorder with the latter also correlating with depression severity. Together, these data suggest primary involvement of SN-RN and DA-VTA in major depressive disorder; this distinguishes major depressive disorder from both bipolar disorder mania and depression as well as schizophrenia where changes in DA-SN rather than in DA-VTA seem to play a prominent role in conjunction with those in SN-RN.

Finally, the subcortical modulation of cortical networks extends beyond the SMN. For instance, both dopamine (DA) and serotonin (SE) modulate the DMN in opposite ways: DA decreases DMN activity (through DA-VTA) while serotonin (through raphe nucleus) increases it.^5^ Together with the opposite effects of DA and SE on SMN (see above), this leads to DA- and SN-based reciprocal modulation of DMN and SMN on the cortical level.^7^ Following their subcortical DA-SN and SE-RN findings (as described above), bipolar mania and depression can be characterized by opposite changes in the DMN–SMN balance: mania exhibits increased SMN activity, e.g. neural variability and decreased DMN variability whereas an opposite pattern with decreased SMN and increased DMN can be observed in bipolar depression.^7^ This entails increased motor activity, e.g. psychomotor agitation co-occurring with decreased internally oriented cognition in mania. While major depressive disorder features the opposite pattern with decreased motor activity, e.g. psychomotor retardation and increased internally oriented cognition (like rumination and increased self-focus).^13^

Together, subcortical biochemical balances between DA and SE modulate cortical topography in a systematic and intrinsically determined way. Subcortical disbalances of DA and SE consequently lead to predictable changes in cortical activity such as SMN and related motor behaviour, e.g. psychomotor agitation or retardation, across different disorders in a symptom-based trans-diagnostic way. Moreover, subcortical disbalances of DA and SE modulate cortical network balances like between SMN and DMN that, in case of their disbalance, lead to predictable symptom pattern, that is, disbalance of internally oriented cognition and psychomotor activity. In sum, we can speak of a biochemical-based subcortical-cortical layer that, through its effects on cortical and mental topography, modulates the co-occurrence of mood, affective, cognitive and psychomotor symptoms.

### From the brain’s topography and dynamics to spatiotemporal psychopathology

In conclusion, we can see that there are predisposed subcortical-cortical patterns that are intrinsic to the brain’s global topography. This concerns the subcortical balance of SE and DA as well as their effects on cortical networks like SMN and DMN whose balance can be traced to and is modulated by the subcortical DA–SE balance. That, in turn, is related to the often observed ‘co occurrence’ of psychopathological symptoms^9^ like psychomotor retardation, low arousal, sadness and cognitive slowing. An outline of these ideas is presented in Fig. 1. Moreover, both subcortical DA–SE balance and subcortical-cortical DA–SE DMN–SMN balance may show high degrees of interindividual variability with some subjects showing stronger modulatory effects of SE while others may exhibit stronger impact of DA. Do these two groups conform to the responders and non-responders in the modelling study? We do not know as detailed clinical information about interindividual differences in symptoms was not provided. Regardless, we can see how the convergence of the modelling approach with a topographic dynamic view of symptoms converge well, strongly supporting what we recently introduced as spatiotemporal psychopathology.^14^ Moreover, this carries major implications for clinical diagnosis and therapy such that psychiatry can finally become a ‘normal’ medical discipline.^15^

**Figure 1:**
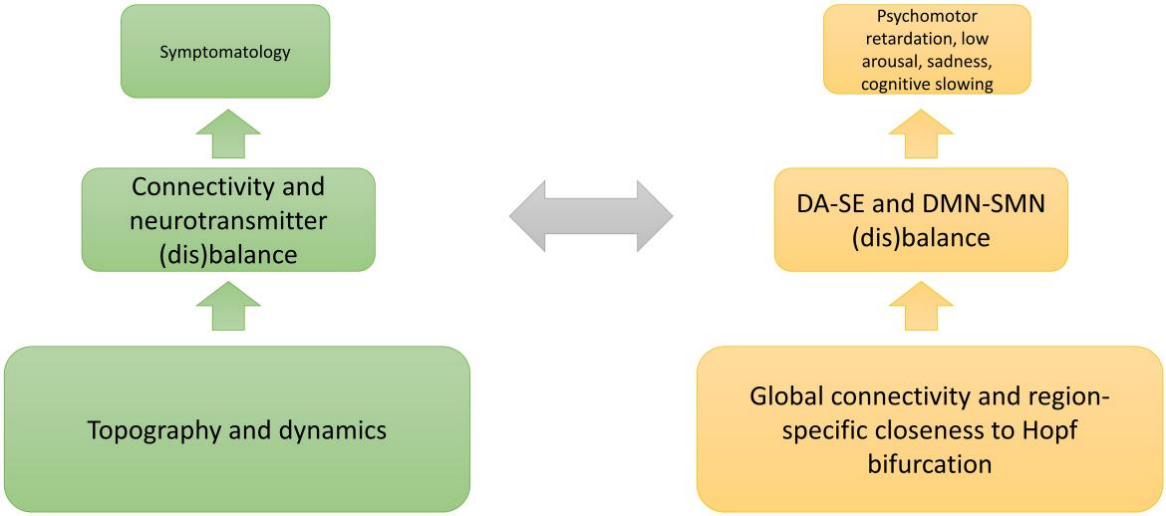
**Topographic and dynamic shaping of symptoms in major depressive disorder.** Abnormalities in topography and dynamics give rise to connectivity and neurotransmitter disbalances, which provides the basis for symptomatology. In the case of major depressive disorder, the disbalances in global connectivity among all regions and closeness to Hopf bifurcations in certain regions can result in DA–SE and DMN–SMN disbalances, which might result in the specific symptomatology in major depressive disorder. DA, dopamine; SE, serotonin; DMN, default mode network; SMN, sensorimotor network.
